# Cognitive Aging and Time Perception: Roles of Bayesian Optimization and Degeneracy

**DOI:** 10.3389/fnagi.2016.00102

**Published:** 2016-05-18

**Authors:** Martine Turgeon, Cindy Lustig, Warren H. Meck

**Affiliations:** ^1^Douglas Mental Health University Institute, McGill UniversityMontreal, QC, Canada; ^2^Department of Psychology, University of MichiganAnn Arbor, MI, USA; ^3^Department of Psychology and Neuroscience, Duke UniversityDurham, NC, USA

**Keywords:** interval timing, attention, clock, memory, decision-making, striatal beat-frequency model

## Abstract

This review outlines the basic psychological and neurobiological processes associated with age-related distortions in timing and time perception in the hundredths of milliseconds-to-minutes range. The difficulty in separating indirect effects of impairments in attention and memory from direct effects on timing mechanisms is addressed. The main premise is that normal aging is commonly associated with increased noise and temporal uncertainty as a result of impairments in attention and memory as well as the possible reduction in the accuracy and precision of a central timing mechanism supported by dopamine-glutamate interactions in cortico-striatal circuits. Pertinent to these findings, potential interventions that may reduce the likelihood of observing age-related declines in timing are discussed. Bayesian optimization models are able to account for the adaptive changes observed in time perception by assuming that older adults are more likely to base their temporal judgments on statistical inferences derived from multiple trials than on a single trial’s clock reading, which is more susceptible to distortion. We propose that the timing functions assigned to the age-sensitive fronto-striatal network can be subserved by other neural networks typically associated with finely-tuned perceptuo-motor adjustments, through degeneracy principles (different structures serving a common function).

## Introduction

We often hear that time flies by as we get older, but that idea is most applicable to our retrospections on years gone by e.g., Gallant et al. (1991), Hinton and Meck (1997a), Ukraintseva (2001), Wearden (2005), Friedman and Janssen (2010), Sucala et al. (2010), Janssen et al. (2013); (cf., McAuley et al., 2006). Our ability to time intervals in the milliseconds-to-minutes and extending into the hours-to-days range of circadian timing (Lewis and Miall, 2009) relies largely on different neural systems (Hinton and Meck, 1997b; Buhusi and Meck, 2005; Buonomano, 2007; Agostino et al., 2011; Hass and Durstewitz, 2016). Age differences in the temporal window of integration and performance on various timing tasks in the milliseconds-to-minutes range are often quite subtle or nonexistent (e.g., Rammsayer et al., 1993; Horváth et al., 2007), and in many cases almost completely accounted for by age differences in other cognitive functions such as attention and working memory (Krampe et al., 2002; Wittmann and Lehnhoff, 2005; Desai, 2007; Ulbrich et al., 2007; Bartholomew et al., 2015) and/or in circadian rhythms (Meck, 1991; Lustig and Meck, 2001; MacDonald et al., 2007; Halberg et al., 2008; Anderson et al., 2014; Golombek et al., 2014). The age differences that do exist have traditionally been explained using an information-processing framework, typically with an attentional gate and/or switch that allows pulses that mark the passage of time to accumulate and be passed to working memory, where they are compared with standard values drawn from reference memory (Meck, 1984; Zakay and Block, 1997; Vanneste and Pouthas, 1999; Vanneste et al., 2001; Lustig, 2003; Allman et al., 2014b).

However, the neurophysiological plausibility of these pacemaker-accumulator models has been called into question (Matell and Meck, 2000; van Rijn et al., 2014). More current timing models instead emphasize the role of neural oscillations in providing the “raw material” analogous to the pulses or ticking of the clock, and the coincidence detection of patterns in those oscillations that mark relevant durations (e.g., Matell and Meck, 2004; Lustig et al., 2005; Allman and Meck, 2012; Merchant et al., 2013). A related development is the proposal that Bayesian processes govern decisions about time when a participant is comparing the current duration to the stored values of some previously timed standard (e.g., Jazayeri and Shadlen, 2010; Cicchini et al., 2012; Shi et al., 2013; Gu et al., 2015a; Shi and Burr, 2016; van Rijn, 2016). To our knowledge, these theoretical proposals have not yet been integrated into the broader literature on aging and time perception. Here, we take the first steps towards such an integration, and address the question of whether age differences in interval timing reflect “nothing more” than age differences in general cognition (especially attention and working memory). Alternatively, there may be fundamental age differences in the quality of timing information that older adults may attempt to compensate for via attention, working memory, as well as increased reliance on environmental support and timing circuitries other than cortico-striatal timing circuits.

The idea that attention influences our perception of time is intuitive and is reflected in popular culture, e.g., “time flies when you’re having fun” but “a watched pot never boils”. In other words, the less attention that is paid to the time dimension, the slower one’s internal clock runs relative to the passage of physical time. This leads to the under-estimation and over-production of intervals relative to physical time (i.e., a person with a slow internal clock may perceive a 5-s stimulus as lasting only 3 s, and when asked to produce a 3-s interval, instead produce a 5-s one). Supporting this intuitive understanding of the relation between attention and time, laboratory studies consistently find that interval-timing performance is highly sensitive to attentional manipulations (e.g., divided attention and distraction) and that timing tasks and other tasks (e.g., memory search) that also load on attention and working memory show mutual interference (e.g., Penney et al., 1998, 2014; Bherer et al., 2007; Brown et al., 2015; Fortin and Schweickert, 2016). Not surprisingly, then, most studies comparing young and older adults on interval-timing tasks find that the presence and size of young adults’ performance advantage depends heavily on attention and memory demands (see review and discussion by Block et al., 1999; Lustig, 2003; Balci et al., 2009; Lustig and Meck, 2009; Szymaszek et al., 2009; Krampe et al., 2010; Bisiacchi and Cona, 2016).

On the other hand, it would be surprising if older adults showed no decline in timing *per se*, as the dopaminergic functions and cortico-striatal circuits that support our sense of time (e.g., Hinton and Meck, 2004; Matell and Meck, 2004; Meck and Malapani, 2004; Lustig et al., 2005; Meck and N’Diaye, 2005; Meck et al., 2008a; Merchant et al., 2013; Agostino and Cheng, 2016) are among the most sensitive to age-related decline (e.g., Rubin, 1999; Raz et al., 2010; Seidler et al., 2010; Abedelahi et al., 2013; Bauer et al., 2015; Kleerekooper et al., 2016). Most reviews emphasize the age differences observed in timing when demands on attention and working memory are high. However, age differences are also found in situations where the opportunity for such processes to make a contribution appears to be quite low. For example, Turgeon and Wing (2012) tested healthy adults across ages 19–98 on a series of unpaced timing tasks where performance relies largely on internal representations and the opportunity to detect and correct errors is relatively low (see also McAuley et al., 2006). These included spontaneous motor tapping (SMT), where the participant is simply told to tap at their most comfortable pace, serial interval production (SIP), where the goal is to tap at a rate of 1 s and $\frac{1}{2}$ s, but no external standard is presented, fastest regular tapping (FRT), where the goal is to tap regularly as quickly as possible, and continuation tapping (CT), where participants first tap in rhythm to an external stimulus, but are then asked to continue tapping at that rate when the stimulus is discontinued. Across all of these tasks, greater age was associated with longer and more variable tapping rates, indicating a slower and more variable internal clock. Additional analyses indicated that these increases were reflective of clock rather than motor variance. In most cases, the increases with age were quite subtle until advanced age (75+ years), suggesting that previous studies failing to find age effects (e.g., Surwillo, 1964; Arenberg, 1968; Salthouse et al., 1979; Block et al., 1998) may have suffered from a lack of power due to smaller participant numbers and older adults who were primarily in the “young-old” (under 75 years) age range. See Kołodziejczyk and Szelsg (2008) for a study of temporal order judgments including centenarians.

In a similar manner, Ramos et al. (2015, 2016) measured tactile temporal discrimination thresholds (TDT) in healthy individuals from 18 to 79 years of age. The TDT was measured as the individual’s ability to discriminate two short (0.2 ms) tactile stimuli from each other as a function of the inter-stimulus interval (ISI). Consequently, the TDT is the shortest ISI that allows a participant to reliably perceive successive stimuli, tested using 6 trials of alternating ascending and descending limits. No effect was observed for gender, race, ethnicity, or handedness, and the reproducibility of the results was good. The overall finding was that every year of increased age was associated with a 0.66 ms increase in TDT. These findings were discussed in terms of models for interval timing involving clock, memory, and decision stages (Allman and Meck, 2012) with the conclusion that age-related effects at the clock stage were most likely to account for the data. The slowing down of an internal clock as a function of age would be expected to lead to the ISI separating the 0.2 ms duration electrical stimulations to be subjectively shorter, making it more difficult to separate sequential stimulus presentations. This finding is of interest given the potential importance of TDT as a behavioral screen for various genetic components of neurological dysfunction (e.g., Conte et al., 2010, 2012, 2014, 2015).

On the one hand, the results of these relatively simple tapping and duration discrimination tasks, which would seem to minimize the involvement of attention and memory processes, seem to point to a “true” age difference in clock speed and time perception. In a seeming paradox, however, there is little evidence that the accuracy and precision of magnitude estimations decline with age on more difficult or “cognitively based” duration discrimination or production tasks once age differences in general intelligence/cognitive function (attention, working memory, resource-sharing, and processing speed) are taken into account (e.g., Salthouse, 1996; Wearden et al., 1997; Greenwood and Parasuraman, 2003; Baudouin et al., 2004; Wearden, 2005; Hancock and Rausch, 2010; Lambrechts et al., 2013; Bartholomew et al., 2015). For example, in the largest timing study to date, evaluating 647 participants, Bartholomew et al. (2015) found that both discrimination and production were strongly correlated with scores on a general cognitive battery; more importantly, after controlling for cognitive scores, timing performance was unrelated to age. However, a potential caveat is that the age range of participants was limited to 18–67 years, precluding the observation of potential aging effects on timing accuracy and precision that may only become evident or independent of cognitive processes sometime after approximately 75 years of age (c.f., Turgeon and Wing, 2012).

These patterns could be explained in two ways. From a “timing-centric” perspective, they may reflect that “timing is everything”, that is, a hallmark of general intelligence and cognitive function that may fundamentally underlie other cognitive functions. This view receives some support from the increasing interest in oscillatory function in general cognition (for reviews, see Siegel et al., 2012; Henry and Herrmann, 2014). Alternatively, timing may depend on the interaction and output of those cognitive functions. Teasing apart these two possibilities is an important challenge for current research, and the explanation may differ depending on the level of analysis. However, in either case, the proposition that interval timing and cognition are intricately linked leads to two predictions. First, the scalar property—reflecting the proportional relationship between the mean of the duration being timed and the standard deviation (SD) of these estimates (i.e., coefficient of variation [CV] is constant)—is the hallmark of interval timing (e.g., Gibbon and Church, 1984a,b; Gibbon et al., 1984; Church, 2003; Buhusi and Meck, 2005; Cui, 2011). This suggests that there should be a similar relationship between sources (e.g., clock, memory, and decision) and forms of variability (e.g., Bernoulli, Gaussian, or Poisson distributions) for other cognitive processes (e.g., Gibbon, 1992; Rakitin et al., 1998, 2005; Cordes et al., 2001; MacDonald and Meck, 2004; Baudouin et al., 2006a,b; Jazayeri and Movshon, 2006; Rakitin and Malapani, 2008; Cordes and Meck, 2014; Namboodiri et al., 2014; Gu et al., 2015b) given the need for the synchronization of oscillatory processes among brain areas for information transfer (e.g., Cheng et al., 2008b; Buehlmann and Deco, 2010; Gu et al., 2015b). Second, from a translational perspective, engaging in mental and physical exercises that require precise timing, balance, and motor coordination during practice (e.g., musical drumming, piano playing, flamenco dancing, video gaming, etc.) should improve not only our sense of time, but also other cognitive processes (e.g., Krampe and Ericsson, 1996; Lustig et al., 2009; Donohue et al., 2010; Anderson et al., 2012, 2013; Cicchini et al., 2012; Kattenstroth et al., 2013; Szabo et al., 2013; Bamidis et al., 2014; Benoit et al., 2014; Szelag and Skolimowska, 2014; Dallal et al., 2015; Kshtriya et al., 2015). Moreover, recent results suggest that rhythmical training exercises can counteract the lengthening errors of total duration in rhythmic reproduction observed after 60 years of age (Iannarilli et al., 2013). Earlier findings, however, provide a note of caution by indicating that moderate levels of skill do not protect against the negative age-related decline in temporal processing and that a certain level of expertise needs to be achieved in order for benefits to be observed (Krampe et al., 2001, 2002; Krampe, 2002).

## Age and Timing Performance: Decline, Preservation, and Compensation

One interpretation of the above statements is that age differences in cognition (e.g., attention, memory, and decision-making) provide more proximal and parsimonious explanations of age differences in timing than does the proposal that older adults generally have a slower internal clock (e.g., Block et al., 1998; Lustig and Meck, 1998, 2001, 2002, 2005, 2011; Lustig, 2003; Bherer et al., 2007; Gooch et al., 2009; cf., Ragot et al., 2002; Pouthas and Perbal, 2004; Moni et al., 2014). Nonetheless, numerous brain areas (e.g., caudate and frontal lobes) tend to atrophy as a consequence of normal aging and the shrinkage of these neural networks is a mediator of reduced dopamine-related temporal processing (e.g., Rubin, 1999; Cheng et al., 2006, 2007; Li et al., 2010; Coull et al., 2011; Gu et al., 2015a). As noted above, the cortico-striatal circuits that support our sense of time (e.g., Matell and Meck, 2004; Lustig et al., 2005; Meck and N’Diaye, 2005; Meck, 2006a,b,c; Meck et al., 2008a; Merchant et al., 2013) are one of the most sensitive neural networks in terms of age-related changes, suggesting that decreases in clock speed *per se* and increases in temporal variability should also be associated with normal aging (e.g., Bäckman et al., 2010; Hurley et al., 2011; Klostermann et al., 2012).

Age-related changes in brain and behavior likely involve long-term dynamic interactions between neural degeneration and recovery processes both within “canonical” regions involved in timing by both young and old adults, possibly involving compensatory sprouting within the damaged pathways (Song and Haber, 2000), buffering of noisy and sustained environmental perturbations (Domijan and Rand, 2011), and compensatory recruitment of other neural networks (Cabeza et al., 2002). This last form of compensation has been referred to as “degeneracy” (Edelman and Gally, 2001; Whitacre, 2010; Whitacre and Bender, 2010), namely, the ability of different brain regions and networks to produce the same or highly similar output, especially when the primary or canonical circuits are dysfunctional or impaired (e.g., Meck, 2002a; Jahanshahi et al., 2010; Lewis and Meck, 2012; Jones and Jahanshahi, 2014, 2015; Harrington and Jahanshahi, 2016). We will use the term “*de-generacy*” as suggested by Mason et al. (2015) to minimize the common association between *degeneracy* and *degeneration*^1^. De-generacy is apparent in many of our neural systems, including vision, hearing, movement, etc. and is distinctive from redundancy in that structurally different mechanisms are involved in the former and multiple copies of identical mechanisms are involved in the latter (Mason, 2010). In the simplest sense, de-generacy can be thought of as a strategy by which the organism protects itself against loss of a vital function by having distribution of function in combination with structural variation. When it comes to purely neural systems, however, de-generacy can be more complex and subtle. It is unlikely that any two separate neural systems perform a given function in exactly the same way unless they have an almost identical neural architecture (as in the examples listed above). Instead, it is becoming increasingly apparent that the brain frequently provides several alternate routes to any given goal, with each of these drawing upon quite separate machinery (Price and Friston, 2002; Brandtstädter and Rothermund, 2003). Evidence for this type of “synergistic replication” comes both from patients with brain damage and from neuroimaging studies of normal, healthy participants. The former group frequently show a remarkable resiliency, still performing well on tasks such as semantic judgment and motor control even when the regions that are most strongly associated with these functions have been fully resected. Similarly, the huge variability in functional imaging results show that different participants perform the same task in different ways (e.g., using different neural systems). These data have been elegantly explained as evidence for de-generacy (Price and Friston, 2002; Noppeney et al., 2004). Furthermore, the proponents of this approach argue that such de-generacy is highly adaptive because it allows flexibility at an evolutionary level: if no single system is 100% essential, then it is more feasible to experimentally alter them without causing fatal side effects (Whitacre, 2010, 2012; Whitacre and Bender, 2010, 2013). The applicability of this line of thought to time perception should be obvious—it is very difficult to completely abolish time perception, especially as a result of focal, unilateral brain lesions where redundancy from the opposite hemisphere is likely to contribute to recovery (Lewis and Meck, 2012). Moreover, it has been proposed that de-generacy occurs as an active monitoring process during sleep, much like the sleep-dependent consolidation of temporal rhythms and other memories (e.g., Cheng et al., 2008b, 2009; Soshi et al., 2010; Lewis et al., 2011; Lewis and Meck, 2012; Scullin and Bliwise, 2015).

Regardless of the proximal cause of aging (Blagosklonny, 2012), de-generacy could explain why timing dysfunctions are likely to be less obvious in normal aging and/or during the initial stages of neurodegenerative disorders such as Parkinson’s and Huntington’s diseases than in experimental subjects with targeted bilateral brain lesions or genetic manipulations (e.g., Liu et al., 2002; Meck and Benson, 2002; Meck, 2006a,b; Desai, 2007; Centonze et al., 2008; Wild-Wall et al., 2008; Balci et al., 2009; Meck et al., 2012a; Church et al., 2014). That is, older adults (and these other patient populations) may be able to recruit alternative cognitive processes and neural networks to maintain performance, at least until those alternatives also “break down” under cognitive demand or due to age-related (or disease-related) physical declines (e.g., Paulsen et al., 2004). This would also explain the seeming paradox that on the one hand, age differences are quite reliable on the simplest timing tasks that attempt to minimize cognitive involvement (i.e., there is little or no opportunity for these alternative processes/networks to intervene), but on the other hand, once one enters the “cognitive realm”, age differences tend to increase with demands on functions such as attention and working memory (i.e., the task demands eventually exceed the ability of older adults to compensate). Interestingly, the effects of de-generacy and the application of a Bayesian decision rule would lead to the “migration” of temporal memories towards each other and violation of the scalar property of interval timing whereby longer durations are timed with less variability than shorter durations (e.g., Malapani et al., 1998; Rakitin et al., 2006; Shi et al., 2013; Gu et al., 2015a).

**Figure 1:**
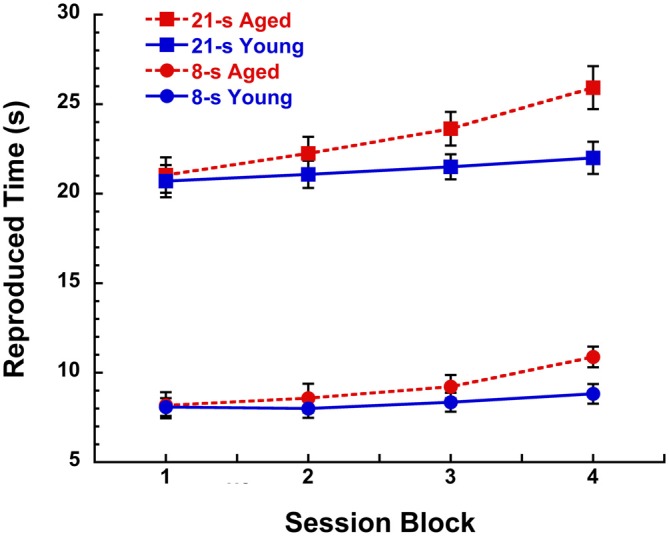
**Reproduced time (s) in a peak-interval procedure with 8-s and 21-s target durations for young (19–28 years, *n* = 7) and aged (67–90 years, *n* = 12) participants as a function of 30 min blocks during a 2 h session without feedback.** Participants received training with inter-trial interval feedback prior to the discontinuation of feedback during the test phase. Data are taken from test sessions described by Malapani et al. (1998) and Rakitin et al. (1998). See Lustig and Meck (2005), Lake and Meck (2013), and Yin et al. (2016a) for a review of the benefits of the peak-interval procedure.

As we grow older, the speed of our internal clock seemingly winds down over the course of a day and take longer to recover than when we were younger. This “fatigue effect” has been proposed to be the result of the gradual depletion of striatal dopamine as a function of sustained cognitive engagement during skill learning acquisition (Kawashima et al., 2012) and is facilitated by certain dopamine-related disorders such as normal aging, adult attention deficit hyperactivity disorder, and Parkinson’s and Huntington’s diseases (e.g., Malapani et al., 1998; Meck, 2005; Balci et al., 2009; Allman and Meck, 2012; Gu et al., 2015a). This more rapid depletion in dopamine function is accompanied by our sense that the external world is going faster, when in fact it may be our internal clock that is going slower, thereby suggesting to us that sequences of events are occurring in a shorter amount of time than would typically be expected (see Cooper and Erickson, 2002). An example of this “fatigue effect” was reported by Malapani et al. (1998) in their study of Parkinson’s disease patients and aged-matched controls trained and then tested on a duration reproduction procedure without feedback (see Yin et al., 2016a for procedural details). Over the course of a 2 h session, healthy aged participants showed proportional rightward shifts in the reproduction of 8-s and 21-s target durations that increased as a function of 30-min session blocks as illustrated in Figure 1. In contrast, young participants (Rakitin et al., 1998) demonstrated a much smaller trend that didn’t reach significance. This relative discrepancy between physical and psychological measurement is what most influences our sense of time in every day life (e.g., McAuley et al., 2006; Matthews et al., 2014; Wearden et al., 2014; Matthews and Meck, 2016).

Like memory and intelligence, our sense of time is multifaceted and some timing abilities are likely more resilient to the aging process than others. Timing tasks that do not involve controlled attention and working memory, or at least do so with a very minimal load (e.g., remembering the current task instructions like “tapping every second”) are more indicative of our ability to perceive and produce temporal intervals than those that do (e.g., reproducing a series of intervals of a complex rhythm in the correct order). Even within less cognitively demanding (or low level) timing tasks, there is substantial heterogeneity in how performance varies with age. Results from paced and unpaced tapping tasks, respectively reported in Turgeon et al. (2011) and Turgeon and Wing (2012), demonstrate that timing error detection and correction abilities are preserved into advanced age, despite a reduction in timekeeping abilities. Indeed, individuals in their 8th and 9th decades of age were as sensitive to the presence of unpredictable temporal perturbations (a sound happening slightly later than it should have were the sequences completely regular, the rhythm being preserved from that point on) as those in their 3rd decade of age. Specifically, the just detectable phase shifts varied from 5 to 15% of Inter-Onset Interval (IOI) across individuals independently of age.

Weber fractions were estimated for intervals spanning a wide portion of the pulsation zone (i.e., IOIs of 300, 600 and 900 ms), that is, intervals that while repeated evoke the sensation of a pulse (or rhythm) and a tendency to move in unison with it (i.e., entrainment). Hence, the high sensitivity to deviations from regularity in old age is likely to reflect adjustments in motor preparedness (i.e., when to perform the next move). Indeed, when tapping in time with the sounds of sequences containing such unpredictable perturbations, elderly participants were as efficient in adjusting for the timing of their taps (i.e., within 1–3 tone(s) from the phase-shift location) as their young counterparts; whether these errors were consciously detectable or not. This suggests that predictable timing mechanisms, that is, the neural processes allowing an individual to generate accurate predictions as to when the next event or series of events should happen and adjust behavior accordingly, are quite resilient to the aging process (cf., Bornkessel-Schlesewsky et al., 2015). In contrast, the same elderly individuals are more variable in tasks depending mainly on the integrity of an internal clock, like tapping regularly as fast as possible (fastest regular rate of FRT) or at the most comfortable rate referred to as SMT.

Other timing tasks showing a clear increase in variability with age were: SIP and the continuation part of synchronization-continuation (SC). While SIP involved tapping every second or half of a second (i.e., twice as fast), SC requires tapping at a regular rate after the pacing sequence ends at the same rates as sensorimotor synchronization (SMS) task, i.e., IOIs of 300, 600 and 900 ms. Moreover, the age-related increased variability in CT could not be attributed to motoric factors (see Figure 5 in Turgeon and Wing, 2012). Interestingly, even though elderly participants tended to produce longer intervals when asked to tap every second or every half second (i.e., age-related impairment in timing accuracy), relative timing accuracy did not decline with age, that is, they produced taps twice as fast for the 0.5-s target interval as for the preceding 1-s one to the same degree as young participants. This is consistent with the use of the just-produced slow sequences as a baseline reference for the adjustment of the ongoing “twice-as-fast” rate. Accordingly the initial 1-s SIP trials are more likely to reflect an internal representation of an interval of 1 s than the subsequent 0.5-s SIP trials in which the just-produced series of slower taps serve as highly useful external cues forming a 1–2 ratio with the current target interval of half a second. These results are in general agreement with the use of all available contextual information (in this case that of concurrent and/or previous event sequences) to improve precision (reduce temporal uncertainty) as predicted by Bayesian models of interval timing (Shi et al., 2013; Gu et al., 2015a). As we will discuss below, Bayesian optimization models can be fruitfully integrated with more general theories of neurocognitive aging in order to provide a new perspective on when (and how) age differences in timing performance are and are not likely to be observed (De Ridder et al., 2014).

## A Bayesian Decision Theory Perspective

The idea of increased noise and variability in older adults coordinates with Bayesian decision theory to explain patterns of age differences and preservation in timing performance. From this perspective, the proposal would be that older adults build an internal representation of both the experimentally imposed distribution of signal durations (the *prior*) and of the error (the *loss function*). This means that when participants are asked to reproduce or compare a recently presented signal duration they incorporate the knowledge of the distribution of previous durations into the perception of the current duration, thus biasing the reproduction towards the mean of the distribution of all durations experienced within a particular context (e.g., Jazayeri and Shadlen, 2010; Acerbi et al., 2012; Cicchini et al., 2012; Gu et al., 2015a). In Bayesian models of this sort, it is hypothesized that a tradeoff exists between accuracy and precision, such that the distribution of durations within a particular context is used to optimize timing performance by reducing uncertainty at the cost of accuracy. In this case, the implicit knowledge of the underlying distribution of durations from which a sample is drawn would be useful when the current clock reading is unreliable due to the effects of variability which may result from age-related declines in dopaminergic function and clock speed (e.g., Malapani et al., 1998; Lake and Meck, 2013; Gu et al., 2015a; Cheng et al., 2016). This explains how the inter-mixing of the memories of previous trials signal durations with the current trial’s clock reading could bias performance (Penney et al., 1998; Gu and Kukreja, 2011; Gu and Meck, 2011; Matell and Kurti, 2014; van Rijn et al., 2014). Under challenging or stressful conditions, however, this statistical analysis provides an efficient strategy for reducing variability in the presence of high levels of uncertainty that may accompany age-related declines in temporal processing (Gu and Kukreja, 2011; Shi et al., 2013; Gu et al., 2015a). In order to justify such an approach, however, one must first demonstrate that older adults do in fact have a slower and/or more variable internal clock. Unfortunately, previous applications of Bayesian decision models have made this assumption without providing direct evidence of such effects (e.g., Sato and Aihara, 2011; Gu et al., 2015a).

## A Slower/Noiser Clock: Direct Evidence in Support of a Bayesian Optimization Approach to Age Differences in Timing

**Figure 2:**
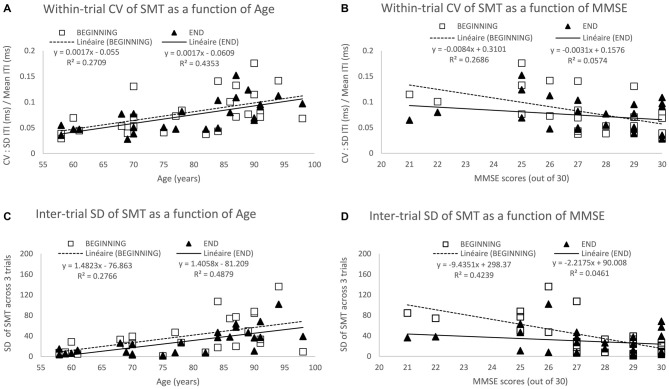
**Spontaneous motor tempo (SMT) across age and its associated variability at the beginning and end of testing.** The top two panels show the mean coefficient of variation (CV) across the first 3 trials (beginning) and the last 3 trials (end), each CV equaling to the standard deviation (SD) of inter-tap intervals (ITI) divided by its mean on a given 30-s trial) across Age **(A)** and mini mental state examination (MMSE) scores **(B)**. The bottom two panels show the SD of the mean ITI across the 3 trials at the beginning and end of the study across Age **(C)** and MMSE scores **(D)**. Data represent additional analyses of the findings reported in Turgeon and Wing (2012).

Regular tapping at the most comfortable rate (i.e., SMT procedures) has been assumed to reflect the natural resonance (or referent) period of the internal clock (McAuley et al., 2006). Variability in SMT is thus a good indicator of how noisy the clock becomes with age. When asked to tap at a rate that feels comfortable and natural for 30 s, older people produced longer inter-tap intervals (ITI) than younger people (see Figure 2A—Turgeon and Wing, 2012); this is consistent with a slowing of their internal clock (Vanneste et al., 2001). In addition, age was associated with relatively large increases in variability. Compared to the variability in tapping performance for the 15 youngest participants (age 19–30 yrs) who were quite stable (*SD* = 65 ms or 12% of mean of 549 ms), those produced across the 15 eldest participants (age 78–98 yrs) varied almost twice as much (*SD* = 177 ms or 21% of mean of 839 ms).

Within-trial variability as measured by the CV for each of the 60 participants (3 trials at the beginning and 3 trials at the end of the study) increased with age (see Figure 2B—Turgeon and Wing, 2012), age accounting for 13% and 11% of the variance among CV scores at the beginning and end, respectively. As most of the age-related changes observed in this study occurred at the top end of the age spectrum, we performed a more focused analysis for the 26 participants aged between 58–98 years for whom we have measures of general cognitive abilities using the Mini Mental State Examination (MMSE — Folstein et al., 1975). This additional analysis of the Turgeon and Wing (2012) data allowed for the examination of both the within-trial CV and the inter-trial SD as a function of age and MMSE at the beginning of the study when participants performed without practice and at the end of testing after having completed a variety of unpaced and paced tapping tasks. The results from these additional analyses (as illustrated in Figure 2) provide further evidence for a noisier internal clock in older adults, even when taking into account general cognitive abilities (MMSE scores) and practice effects (beginning vs. end of study measures).

Notably, even in this supposedly minimally-cognitive timing task, the effects of attention and memory can be seen and may interact with age. Although the MMSE is a rough measure of general cognitive abilities, the score accounts for a substantial proportion of the variance at the beginning of testing, namely 27% of the variance among within-trial CV (Figure 2B) and 48% among inter-trial SD (Figure 2D). In contrast, MMSE no longer predicts variability within or across trials at the end of testing, only 5–6% of the variance being accounted for by the MMSE. That general cognitive abilities contribute to a reduction in variability on the very first task seems plausible as older adults with high MMSE scores are more likely to understand the instructions rapidly, while those with lower MMSE scores might require a bit longer to “get into the swing” of tapping tasks. For instance, they might need to be reminded to be as regular as possible and maintain the same pace for the whole half-a-minute period. The lack of a relationship between MMSE and variability at the end of testing suggests that practice overcame any initial cognitive challenges of the SMT task. Interestingly, the predictive effect of Age on within-trial CV (Figure 2A) and inter-trial-SD (Figure 2C) is actually higher at the end (43–48% of variance accounted for) than at the beginning (27–28% of variance accounted for). This suggests that despite the age-related decline in performance due to general cognitive factors, there is an increased variability within and across trials arising from slower and less reliable timing mechanisms consistent with our earlier discussion of dopamine depletion and cognitive “fatigue effect” across a session. On a methodological front, these findings point to the importance of including enough trials and analyzing performance only after it has stabilized to increase the likelihood of testing “true” age differences in timing while minimizing artifacts and confounds due to individual or group differences in task acquisition and learning.

**Figure 3:**
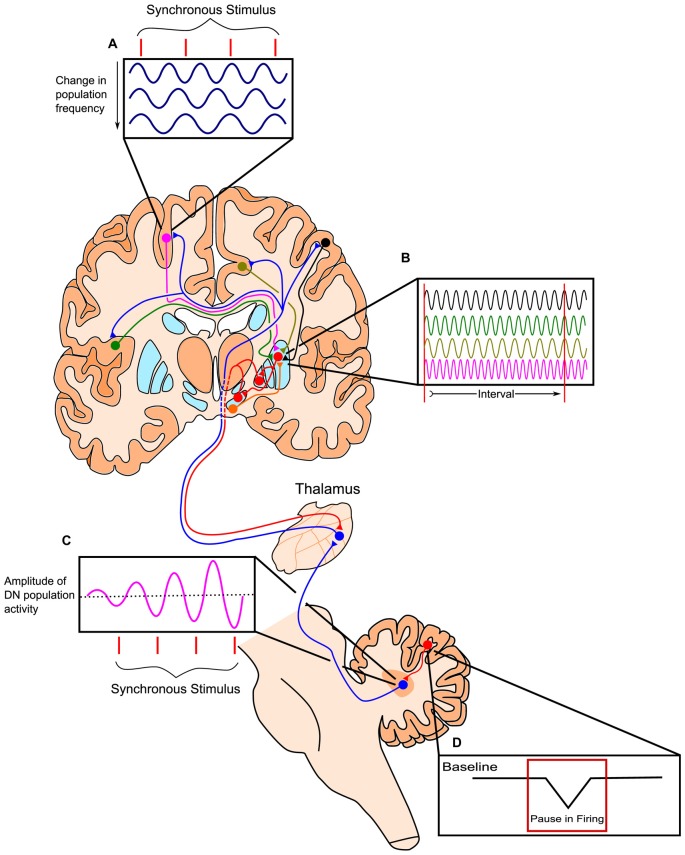
**Outline of the Striatal Beat-Frequency (SBF) model of interval timing with the incorporation of a cerebellar adjustment mechanism into the time code. (A)** At the start of a to-be-timed signal a phasic pulse of dopamine from the ventral tegmental area (VTA) synchronizes cortical oscillations. Cortical oscillations in areas such as the prefrontal cortex (PFC) can be modulated with synchronous stimuli possibly through efferents from the thalamus. **(B)** These dispersed cortical neurons synapse onto medium spiny neurons (MSNs) within the striatum, which are activated at specific target durations based on the oscillatory activity pattern of synapsing projections. These neurons project from non-motor regions in the thalamus such as the caudal portion of ventralis lateralis, pars caudalis (VLcc) and receive extensive inputs from the cerebellar dentate nucleus (DN). **(C)** The DN also exhibits changes in population activity in response to synchronous stimuli, which may drive the modulation seen in cortical regions of the cerebrum. **(D)** The change in DN activity is likely modulated by decreases in tonic Purkinje cell activity (pauses) allowing for the precise tuning of timing mechanisms through disinhibition. Adapted from the initiation, continuation, adjustment, and termination (ICAT) model of temporal integration described by Lusk et al. (2016) and Petter et al. (submitted).

The age-related increase in inter-trial variability in combination with the age-related decrease in precision (i.e., increase of CV with age) on SMT as well as other unpaced tapping tasks reported by Turgeon and Wing (2012) is *consistent with a noisier and less reliable clock in older participants with otherwise intact predictive timing mechanisms*—as assessed from error detection and correction performance, with the external sound sequence providing some “feedback” that reduces uncertainty. The fact that age does not predict relative timing accuracy in SIP (see Figure 3C—Turgeon and Wing, 2012) provides further evidence that when a temporal context is available (i.e., a target period with a simple 2:1 ratio in SIP or a pacing sequence in SMS), it is used to compensate for a slower, more variable internal clock in an aging nervous system as opposed to effects on motor variability (e.g., Wing and Kristofferson, 1973).

Procedural limitations (e.g., limited range of durations and the use of magnitude estimation or reproduction procedures allowing for the calibration and rescaling of stimulus durations) as well as inadequate statistical power and improper control for general intelligence factors may have precluded the observation of age-related decreases in clock speed in numerous reports (e.g., Surwillo, 1964; Arenberg, 1968; Salthouse et al., 1979; Block et al., 1998). In general, the observation of age-related rightward horizontal shifts in psychometric timing functions is consistent with the internal clock slowing down if trial-by-trial and/or session-by-session analyses show appropriate temporal dynamics as a function of within-session feedback (e.g., Vanneste et al., 2001; Lustig and Meck, 2005; Rakitin and Malapani, 2008) and/or between-session train and test conditions (e.g., Meck, 1983, 1996, 2005; Malapani et al., 1998; Lake and Meck, 2013). Moreover, the observed reversals in duration categorization around the point of subjective equality for pairs of anchor durations and larger modality differences would be expected to occur with age-related decreases in clock speed as a result of duration discriminations becoming more difficult with a slower, less precise clock that normally exhibits differential sensitivity to auditory and visual stimuli (e.g., Penney et al., 1998, 2000, 2005; Cheng et al., 2008a, 2011) particulary when timing multiple signal durations concurrently (e.g., Lustig and Meck, 2001, 2002; Buhusi and Meck, 2009a,b; McAuley et al., 2010; Todorov et al., 2014). In contrast, systematic changes in temporal accuracy (e.g., horizontal rightward shifts in psychometric timing functions that are gradually acquired over the course of the lifespan and maintained in the face of corrective feedback) are indicative of age-related decreases in memory storage speed (K*) resulting in proportional increases in the durations stored in long-term memory (e.g., Meck, 1983, 1996, 2002a,b, 2006c; Meck and Church, 1985, 1987; Meck et al., 1986; Meck and Williams, 1997; Lejeune et al., 1998; McCormack et al., 1999, 2002; Lustig, 2003; Meck et al., 2008b; Balci et al., 2009; Oprisan and Buhusi, 2011).

In summary, the findings reviewed here lend support to the application of Bayesian models of optimization in order to account for decision-making made under increased levels of uncertainty in the aging brain as a function of age and cognitive fatigue. Moreover, it appears justified to assume a slower and/or noiser internal clock as a contributing factor to this uncertainly above and beyond any age-related changes in attention and memory.

## Striatal Beat-Frequency (SBF) Model: Neurobiological Basis for Bayesian Timing

The striatal beat frequency (SBF) model of interval timing accounts well for much of the pharmacological, neurophysiological, and psychological data on timing and time perception (e.g., Matell and Meck, 2000, 2004; Coull et al., 2011; Oprisan and Buhusi, 2011, 2013, 2014; van Rijn et al., 2011; Allman and Meck, 2012; Buhusi and Oprisan, 2013; Oprisan et al., 2014; Kononowicz, 2015; Kononowicz and van Wassenhove, 2016). The SBF model proposes that time perception is largely subserved by connections between the striatum, cortex, and thalamus, with the dorsal striatum being specifically crucial for proper timing abilities (Meck, 2006a,b). According to this model, the start signal to time a stimulus is marked by the phasic release of dopamine from dopaminergic midbrain projections to the cortex and dorsal striatum (Matell and Meck, 2004; Gu et al., 2011). This neurotransmitter release causes oscillatory cortical neurons to synchronize their firing and resets activity in the dorsal striatum. Thousands of these oscillating cortical neurons converge on individual medium spiny neurons (MSNs) in the striatum. As ensembles of cortical glutamatergic pyramidal neurons oscillate with varying intrinsic frequencies, their oscillations fall out of phase after the initial synchronizing action of dopamine. The different cortical oscillation frequencies result in input activation patterns to striatal neurons that vary with the time elapsed from the cortical synchronization event (e.g., van Rijn et al., 2014; Gu et al., 2015b; Hashimoto and Yotsumoto, 2015; Murai et al., 2016). Each MSN in the striatum is thought to integrate these oscillatory cortical inputs and respond to select patterns of cortical neuronal firing, based on previous reinforcement through long-term potentiation (LTP). In the striatum, cortical firing results in long-term depression, unless there is a concurrent release of dopamine in which case LTP may occur. This dopaminergic input, originating from the dorsal midbrain, and the LTP it induces along this pathway, may strengthen connections with cortical inputs active at the time of reinforcement or feedback. In this way, striatal neurons may become specialized in responding to specific temporal intervals, as the threshold for firing is reduced when the correct cortical inputs are present. Prior to learning, the delivery of an unexpected reinforcement or feedback causes a phasic surge of dopamine release in the striatum that may represent the dopaminergic input necessary for LTP. Striatal output influences activity of the thalamus via a direct and an indirect pathway, which have opposing effects on thalamic activity. In turn, the thalamus has excitatory projections to the cortex, which then project back to the striatum, completing the cortico-thalamic-striatal loop (Buhusi and Meck, 2005; Agostino et al., 2011). The direct and indirect pathways of the basal ganglia are suggested to play a role in the start, stop, and resetting of the timing process, though further research is necessary to elaborate the proposed roles of these pathways in anticipatory timing, intertemporal choice, and temporal discounting (Wiener et al., 2008; Kim and Zauberman, 2009; Cui, 2011; Löckenhoff et al., 2011; MacDonald et al., 2012; Agostino et al., 2013; Heilbronner and Meck, 2014). In addition to cortico-striatal circuits, cortico-cerebellar circuits provide feedback and fine tuning of the processes described above for the cortico-striatal circuits as illustrated in Figure 3 (see Cheng et al., 2016; Lusk et al., 2016; Petter et al., submitted).

As described above, the SBF model provides the necessary neurobiological substrates and neural firing properties in order to identify sources and forms for increased variability associated with aging effects on timing and time perception (e.g., Meck and Malapani, 2004; Buhusi and Oprisan, 2013; Oprisan and Buhusi, 2014; Cheng et al., 2016). For example, a slower clock with increased variability in clock speed could be accounted for by the effects of tonic and phasic dopamine release from the ventral tegmental area (VTA) to the frontal cortex. This would involve specific changes in the period, variability, resetting, and phase of cortical oscillatory processes being monitored by striatal median spiny neurons in the dorsal striatum (e.g., Oprisan and Buhusi, 2011; Gu et al., 2015b; Kononowicz, 2015; Cheng et al., 2016; Kononowicz and van Wassenhove, 2016).

## De-Generacy in Predictive Timing Mechanisms as a Potentail Route for Compensation

Though not specifically addressed by the SBF model, diminished striatal functioning might also engage brain circuits that compensate via de-generacy mechanisms (Lewis and Meck, 2012), wherein different anatomical networks are engaged (Meck, 2002a; Merchant et al., 2013). De-generacy in timing systems is plausible since time-related cell activity is not only found in the basal ganglia, but also the cerebellum, thalamus, posterior parietal cortex, prefrontal cortex (PFC), and the supplementary motor area (SMA and preSMA; Merchant et al., 2013; Strenziok et al., 2013; Lusk et al., 2016; Petter et al., submitted). Timing in different behavioral contexts is also associated with different neural architectures. For example, explicit timing is thought to depend on the striatum and basal ganglia, whereas activation of the SMA, inferior cortex, and cerebellum is considered to be more task specific. In contrast, implicit perceptual timing is associated with a different set of brain regions, most frequently the parietal cortex (e.g., Coull and Nobre, 2008; Coull et al., 2011)—although the cerebellum has been argued to be involved in both explicit and implicit motor timing as well as explicit and implicit perceptual timing, perhaps distinguished from the striatum along a discrete vs. dynamic or duration-based vs. beat-based dimension (e.g., Liverence and Scholl, 2012; Teki et al., 2012; Breska and Ivry, 2016; Petter et al., submitted). Similarly, cortico-cortical systems are more engaged when timed movements are externally paced, whereas the striatum is engaged when movements are self-paced or internally timed (Taniwaki et al., 2003).

The results reported by Turgeon and colleagues (Turgeon et al., 2011; Turgeon and Wing, 2012) provide evidence that the adjustment component of timing is preserved up until the 9th decade of age in healthy, aged brains, that is, those without any diagnosed pathologies. By *adjustment timing*, we mean the adaptive use of predictable intensity fluctuations or temporal dynamics (e.g., the abrupt rise of intensity or onsets of the regularly-spaced events of a metronome) to initiate an action (e.g., when to jump) or series of actions (e.g., when to switch directions when running an obstacle course) and/or regulate an ongoing act/behavior (e.g., in a jazz performance, a double bass player delaying the plucking of a chord following a missed beat by the drummer). Predictability in temporal dynamics is inherent to rhythmic patterns (beat-based timing); however, it is also present whenever the signal provides enough information to generate expectations as to *what should happen when* (perceived and/or produced events). For instance, well-trained contemporary musicians or dancers can learn complex arrhythmic patterns; that is, they can prepare, initiate and smoothly execute the right moves at the right time despite the lack of metrical structure (present in most western music) or non-metrical regularity (as in speech prosody). Adjustment can also be made in the timekeeper’s settings. For instance, the detection of a phase shift in an otherwise perfectly regular sequence presumably leads to a resetting of the clock period, even if no external movement is produced. Of course, the correct detection of temporal perturbations is not informative *per se* for the underlying internal timekeeping parameters. However, the fact that the same participants with the same sequences correct for these errors rapidly when asked to tap in time with the sounds of the pacing sequence is a strong indicator of a resetting of the period of the internal clock following the detection of a phase-shift perturbation. It’s important to note, however, that apart from the age-related effects observed for auditory duration discrimination, the effects of variable rhythmic grouping on temporal sensitivity is greatest among older listeners independent of hearing loss. Such findings have implications for speech discrimination in degraded/noisy environments in terms of identifying deficits in temporal processing that are unrelated to the loss of hearing sensitivity associated with normal aging (Gordon-Salant et al., 2011; Fitzgibbons and Gordon-Salant, 2015).

## Concluding Remarks

Our review of the literature suggests that there are fundamental age-related changes in the functioning of the cortico-thalamic-basal ganglia circuits that implement timing in the hundredths of milliseconds-to-minutes range. There are also instances of at least partial compensation that can in many cases mask age-related declines in timing and time perception and allow older adults to perform as well or nearly as well as young adults until the load of either cognitive demands or physical decline pushes them past their threshold for being able to compensate. Reuter-Lorenz and colleagues (e.g., Reuter-Lorenz and Lustig, 2005; Reuter-Lorenz and Cappell, 2008; Lustig et al., 2009; Lustig and Jantz, 2015) have referred to this as the compensation-related utilization of neural circuits (CRUNCH) hypothesis. Despite age-related declines in cognitive functions such as attention and working memory, older adults are still able to rely on these processes by recruiting additional cognitive resources and capitalizing on the availability of external cues that serve as environmental support. This leads to an increased reliance on predictive timing circuits, monitoring deviations from expectations (i.e., temporal errors) and allowing for adaptive corrections (i.e., online adjustments) to the parameters of internal timekeeping mechanisms and/or external movements like the olivocerebellar and parietofrontal networks (e.g., Turgeon and Wing, 2012; Gu et al., 2015a; Petter et al., submitted).

As a consequence of the above observations, we propose that: (1) as the functioning of MSNs in cortico-thalamic-basal ganglia circuits serving as coincidence detectors of patterns of cortical oscillations become more variable and therefore less reliable with age (see Allman and Meck, 2012), cortico-cerebellar or hippocampal regions that are less affected by the aging process are recruited to influence and/or take over some of these timing functions through de-generacy principles (e.g., Meck, 2002a; Merchant et al., 2013; Lusk et al., 2016; Petter et al., submitted); (2) the dynamic adjustments performed by error correction pathways implement Bayesian optimization principles, namely to estimate the likelihood of an actual event distribution (*prior* function) with as much relevant data as possible and to minimize error (*loss* function), that is the disparity between predicted (via internal clock) and actual (via external feedback) interval series (e.g., Jazayeri and Movshon, 2006; Jazayeri and Shadlen, 2010; Shi et al., 2013; Gu et al., 2015a).

It is important to note that the interplay among the regulation of multisensory integration, clock speed, feedback, and brain dopamine levels that contributes to distortions and preservations in time perception and timed performance are relevant not only to normal aging, but also to the timing differences associated with psychosis, dementia, and other types of neurodegeneration (e.g., MacDonald and Meck, 2005; Meck, 2005; Bonnot et al., 2011; Allman and Meck, 2012; Lake and Meck, 2013; Piras et al., 2014; Gu et al., 2015a; Bedard and Barnett-Cowan, 2016). The overall conclusion is that normal aging is commonly associated with reductions in the speed and increased variability in the operation of a core timing circuit supported by distributed dopamine-glutamate and GABA interactions in cortico-striatal circuits (e.g., Buhusi and Meck, 2002; Tseng and O’Donnell, 2004; Cheng et al., 2006, 2007, 2016; Merchant et al., 2013; Matthews et al., 2014; Terhune et al., 2014). These age-related changes in interval timing and relative time-sharing function at the level of multiple time scales, systematically affecting reaction time and unpaced finger tapping, the playing of sports and musical instruments, consciousness, retrospective and prospective memory processes, and other types of time, number, and reward-based decision making (e.g., Tulving, 2002; Fortin, 2003; Zakay and Block, 2004; Buonomano, 2007; Buhusi and Meck, 2009a; Fortin et al., 2009; Nyberg et al., 2010; Meck et al., 2012b; Turgeon and Wing, 2012; Aagten-Murphy et al., 2014; Allman et al., 2014a; Bermudez and Schultz, 2014; French et al., 2014; MacDonald, 2014; MacDonald et al., 2014; Wolkorte et al., 2014; Zakay, 2014; Yin et al., 2016b).

Given that senescence is observed in natural populations of animals—affecting their foraging strategies based, in part, on interval timing and the setting of temporal horizons (Bateson, 2003; MacDonald et al., 2007), the understanding of age-related changes in timing and time perception would appear to have widespread implications for bio-gerontology, emotional regulation, time-based prospective memory and other types of temporal cognition (e.g., Löckenhoff and Carstensen, 2007; Löckenhoff, 2011; Alexander et al., 2012; Nussey et al., 2013; Anderson et al., 2014; Fingelkurts and Fingelkurts, 2014; Matthews and Meck, 2014, 2016; Tucci et al., 2014; Vanneste et al., 2015; Lake, 2016; Lake et al., 2016; Mather, 2016). By providing a foundation for evaluating brain aging effects on timing and time perception we are now better prepared to evaluate the need for and effectiveness of interventions designed to alleviate age-related declines in temporal cognition (Roberts and Allen, 2016).

## Author Contributions

MT, CL, and WHM jointly wrote the review article.

## Conflict of Interest Statement

The authors declare that the research was conducted in the absence of any commercial or financial relationships that could be construed as a potential conflict of interest.

## Footnotes

^1^In neural Darwinism (Edelman and Gally, 2001), the term *degeneracy* refers to the concept of heteromorphic isofunctionality (i.e., different structures subserving the same function). On the other hand, *degeneration* is a term much more familiar to the lay public; it refers to deleterious decay, including that of neural structures. In a seemingly paradoxical way, de-generacy is a highly adaptive feature of complex biological systems like the brain given the inevitable degenerative processes (i.e., degeneration at many levels of neural organization) that ensues with their aging. Unlike *redundancy* (same structures subserving same functions), the structural diversity arising from de-generacy in the nervous system is highly conducive to functional plasticity or pluripotentiality (one structure subserving different functions). In Mason’s words: “A key corollary of degeneracy is that, because it entails diversity at the structural level, different circumstances may elicit different outputs from the same degenerate set. Thus, one-to-many structure-function relationship has been dubbed *pluripotentiality*” (Mason, 2015). In sum, de-generacy (many-to-one structure-function relationship) is as essential for the emergence of complexity through evolution, and by extension the brain, as is pluripotentiality.
